# AI for social good: unlocking the opportunity for positive impact

**DOI:** 10.1038/s41467-020-15871-z

**Published:** 2020-05-18

**Authors:** Nenad Tomašev, Julien Cornebise, Frank Hutter, Shakir Mohamed, Angela Picciariello, Bec Connelly, Danielle C. M. Belgrave, Daphne Ezer, Fanny Cachat van der Haert, Frank Mugisha, Gerald Abila, Hiromi Arai, Hisham Almiraat, Julia Proskurnia, Kyle Snyder, Mihoko Otake-Matsuura, Mustafa Othman, Tobias Glasmachers, Wilfried de Wever, Yee Whye Teh, Mohammad Emtiyaz Khan, Ruben De Winne, Tom Schaul, Claudia Clopath

**Affiliations:** 1 DeepMind, London, UK; 2 Department of Computer Science, University College London, London, UK; 3 Department of Computer Science, University of Freiburg, Freiburg, Germany; 4 Bosch Center for Artificial Intelligence, Renningen, Germany; 5 Oxfam GB, Oxford, UK; 6 RNW Media, Hilversum, The Netherlands; 7 Microsoft Research, Cambridge, UK; 8 University of Warwick, Warwick, UK; 9 Alan Turing Institute, London, UK; 10 International Commission of Jurists, Brussels, Belgium; 11 Chemonics International Inc., Kigali, Rwanda; 12 BarefootLaw, Kampala, Uganda; 13 RIKEN Center for AI Project, Tokyo, Japan; 14 Justice and Peace Netherlands, The Hague, The Netherlands; 15 Google, Zurich, Switzerland; 16 Shaqodoon Organization, Hargeisa, Somaliland; 17 Institute for Neural Computation, Ruhr-University Bochum, Bochum, Germany; 18 SEMA, Kampala, Uganda; 19 Humanity Solutions, The Hague, The Netherlands; 20 University of Oxford, Oxford, UK; 21 Oxfam Novib, The Hague, The Netherlands; 22 Department of Bioengineering, Imperial College London, London, UK

**Keywords:** Developing world, Ethics

## Abstract

Advances in machine learning (ML) and artificial intelligence (AI) present an opportunity to build better tools and solutions to help address some of the world’s most pressing challenges, and deliver positive social impact in accordance with the priorities outlined in the United Nations’ 17 Sustainable Development Goals (SDGs). The AI for Social Good (AI4SG) movement aims to establish interdisciplinary partnerships centred around AI applications towards SDGs. We provide a set of guidelines for establishing successful long-term collaborations between AI researchers and application-domain experts, relate them to existing AI4SG projects and identify key opportunities for future AI applications targeted towards social good.

## Abstract (web summary)

The AI for Social Good movement aims to apply AI/ML tools to help in delivering on the United Nations’ sustainable development goals (SDGs). Here, the authors identify the challenges and propose guidelines for designing and implementing successful partnerships between AI researchers and application - domain experts.

## Introduction

The challenges facing our world today have grown in complexity and increasingly require large, coordinated efforts: between countries; and across a broad spectrum of governmental and non-governmental organisations (NGOs) and the communities they serve. These coordinated efforts work towards supporting the Sustainable Development Goals (SDGs)^1^, and there continues to be an important role for technology to support the developmental organisations and efforts active in this field to deliver the highest impact.

Artificial intelligence (AI) and machine learning (ML) have attracted widespread interest in recent years due to a series of high-profile successes. AI has shown success in games and simulations^2,3^, and is being increasingly applied to a wide range of practical problems, including speech recognition^4^ and self-driving cars^5^. These commercial applications often have indirect positive social impact by increasing the availability of information through better search and language-translation tools, providing improved communication services, enabling more efficient transportation, or supporting more personalised healthcare^6^. With this interest come a lot of questions regarding social impact, malicious uses, risks, and governance of these innovations, which are of foremost importance^7,8^.

Targeted applications of AI to the domain of social good have recently come into focus. This field has attracted many actors, including charities like DataKind (established in 2012)^9^, academic programmes such as the Data Science for Social Good (DSSG) programme at the University of Chicago (established in 2013)^10^, the UN Global Pulse Labs^11^, AI for Social Good workshops in conferences such as the 2018 and 2019 NeurIPS conference^12,13^, the 2019 ICML conference^14^ and the 2019 ICLR conference^15^, along with corporate funding programmes such as Google AI for Good Grants^16^, Microsoft AI for Humanity^17^, Mastercard Center for Inclusive Growth and the Rockefeller Foundation’s Data Science for Social Impact^18^, amongst several others.

Results from several recent studies hint at the potential benefits of using AI for social good. Amnesty International and ElementAI demonstrated how AI can be used to help trained human moderators with identifying and quantifying online abuse against women on Twitter^19^. The Makerere University AI research group supported by the UN Pulse Lab Kampala developed automated monitoring of viral cassava disease^20^, and this same group collaborated with Microsoft Research and other academic institutions to set up an electronic agricultural marketplace in Uganda^21^. Satellite imagery was used to help predict poverty^22^ and identify burned-down villages in conflict zones in Darfur^23^, and collaborative efforts between climate and machine learning scientists initiated the field of climate informatics^24,25^ that continues to advance predictive and interpretive tools for climate action. Future improvements in both data infrastructure and AI technology can be expected to lead to an even more diverse set of potential AI4SG applications.

This wealth of projects, sometimes isolated, has led to several meta-initiatives. For example, the Oxford Initiative on AIxSDGs^26^, launched in September 2019, is a curated database of AI projects addressing SDGs that indexes close to 100 projects. Once publicly accessible, it should support a formal study of such projects’ characteristics, success factors, geographical repartition, gaps, and collaborations. Attempts at similar repositories include the ITU AI Repository^27^. Another growing initiative, focused on networking AI4SG and making their blueprints easily accessible and reproducible by anyone, is the AI Commons knowledge hub^28^ backed by 21 supporting organisations and 71 members. These meta-initiatives can help aggregate the experience and transfer knowledge between AI4SG projects, as well as establish connections between teams and organisations with complementary aims.

Despite the optimism, technical and organisational challenges remain that make successful applications of AI/ML hard to deliver within the field and that make it difficult to achieve lasting impact. Some of the issues are deeply ingrained in the tech culture that involves moving fast and breaking things while iterating towards solutions, and a lack of familiarity with the non-technical aspects of the problems^29^. There is also a long history of tech for good, including 30 years of Information and Communication Technology for Development (ICT4D). Not all applications of technology aimed at delivering positive social impact manage to achieve their goals^30^, leaving us with important experiences from which we must learn. Importantly, technology should not be imagined as a solution on its own^31^, outside of the context of its application: it merely aligns with human intent and magnifies human capacity^32^. It is therefore critical to put it in service of application-domain experts early, through deep partnerships with technical experts.

To achieve positive impact, AI solutions need to adhere to ethical principles and both the European Commission^33^ as well as OECD^34^ have put together guidelines for developing innovative and trustworthy AI. Related principles are encoded in the Montreal Declaration for Responsible AI^35^ and the Toronto Declaration^36^. The European Commission states that AI needs to be lawful, ethical and robust, to avoid causing unintended harm. OECD Principles on AI state that AI should be driving inclusive growth and sustainable development; designed so as to respect the rule of law, human rights, democratic values and diversity; transparent, so that people can understand AI outcomes; robust, safe and secure; deployed with accountability, so that organisations can be held responsible for AI systems they develop and use. Proper ethical design and governance of AI systems is a broad research topic of fundamental importance, and has been the focus of institutions and initiatives like the AI Now Institute^37^ and the ACM Conference on Fairness, Accountability and Transparency^38^.

Also, it is important to recognise the interconnectedness of the Sustainable Development Goals (SDGs) and of efforts to achieve them. The UN stresses that each goal needs to be achieved so that no one is left behind. Yet, an intervention with a positive impact on one SDG could be detrimental to another SDG and its targets. Awareness of this interconnectedness should also be a driving principle for fair and inclusive AI for social good: AI applications should aim to maximise a net positive effect on as many SDGs as possible, without causing avoidable harm to other SDGs. Therefore, while being careful to avoid the pitfalls of analysis paralysis^39^, both application-domain experts and AI researchers should aspire to measure the effects, both positive and negative, of their AI for social good applications across the five areas of people, planet, prosperity, peace and partnerships, which are the targets of the sustainable development agenda.

A recent UN report^40^ details how over 30 of its agencies and bodies are working towards integrating AI within their initiatives. According to the report, AI4SG projects need to be approached as a collaborative effort, bringing communities together to carefully assess the complexities of designing AI systems for SDGs. These initiatives should aim to involve NGOs, local authorities, businesses, the academic community, as well as the communities which these efforts support. The report highlights the vast potential of the technology across a wide spectrum of applications, while recognising the need for improving data literacy and a responsible approach to AI research and deployment. Our own efforts to put these considerations into practice have led us to put forward in the next section a set of guidelines with which to approach AI4SG, which we then exemplify with a set of case studies before concluding with a call to action for technical communities and their important role in supporting the success of our social and global goals.

## Guidelines for AI4SG collaborations

To address the challenges involved with setting up successful collaborations between AI researchers and application-domain experts working on SDGs, we facilitated a series of structured multidisciplinary discussions at a dedicated seminar^41^ bringing together experts from both communities to identify key aspects of successful partnerships, and potential obstacles. This process involved setting up focused working groups around key topics and repeatedly coming together to disseminate the results, obtain feedback and discuss within the wider group. We present the conclusions in the form of guidelines to inform future AI4SG initiatives and ground our recommendations in practical examples of successful AI4SG collaborations.

**Table:** 

**G1**	Expectations of what is possible with AI need to be well-grounded.
**G2**	There is value in simple solutions.
**G3**	Applications of AI need to be inclusive and accessible, and reviewed at every stage for ethics and human rights compliance.
**G4**	Goals and use cases should be clear and well-defined.
**G5**	Deep, long-term partnerships are required to solve large problems successfully.
**G6**	Planning needs to align incentives, and factor in the limitations of both communities.
**G7**	Establishing and maintaining trust is key to overcoming organisational barriers.
**G8**	Options for reducing the development cost of AI solutions should be explored.
**G9**	Improving data readiness is key.
**G10**	Data must be processed securely, with utmost respect for human rights and privacy.

These guidelines summarise what we see as key principles for successful AI4SG collaborations and should therefore be applicable across different types of organisations aiming to utilise AI for sustainable development. These guidelines pertain to the overall use of AI technology (**G1**, **G2**, **G3**), applications (**G4**, **G5**, **G6**, **G7**, **G8**) and data handling (**G9**, **G10**). The list is by no means exhaustive and we expect there to be notable differences in how each of the guidelines is implemented in practice, depending on the data readiness of each organisation and the theory of change underpinning the projects. The recent report from the Google AI Impact Challenge identifies NGOs in particular as having a low rate of utilising AI in their existing projects, which is why we feel they might be the ones to benefit the most from the guidelines provided here^42^.

The fast pace of AI research may sometimes make it difficult for organisations outside the field to correctly assess the applicability of the current state of the art. It is therefore important to set expectations early (**G1**), to distinguish between short-term and long-term opportunities and help select projects accordingly.

Despite the apparent appeal of using the latest ML methods, these may require large quantities of high-quality training data. AI4SG projects may sometimes benefit from simpler solutions^43^, aiming to solve the problem at hand with minimum overall complexity (**G2**). Such solutions tend to be faster to implement, easier to maintain, interpret and justify—and are sometimes sufficient to solve valuable practical problems, as demonstrated by the winning solution in a recent food safety predictive challenge^44^. Data analysis and visualisation can be a useful tool in informing practical decision-making and can potentially deliver value to organisations.

AI systems need to be fair, inclusive and accessible (**G3**). Fairness in particular should be explicitly accounted for, to avoid reinforcing existing societal biases reflected in the data used for model development^45–47^. Unfairness may result in violations of the right to equality, manifesting as inequity in model performance and associated outcomes across race, ethnicity, age, gender, etc. Fairness of AI applications should be deeply anchored in existing international human rights standards, guiding all practical decisions. Ethics compliance should be appropriately formalised and involve setting up internal and external processes to review sensitive decisions and design choices.

To be actionable, practical problems need to be translated into concrete, well-defined goals (**G4**) that can be addressed by technical solutions. For example, water shortages in case of drought could be addressed by a use case of predicting water demand based on flow data^48^. Alternatively, one could approach the problem by providing better weather predictions or tracking water supply and reservoir levels or helping individual consumers reduce their daily water usage. For each goal, it is crucial to provide the correct metric for measuring the desired effect, as well as define the minimum viable performance for a solution to be adding value to its stakeholders.

We recognise the critical role of focused short-term initiatives like workshops and hackathons in gathering momentum and bringing application-domain experts together with AI researchers to deepen their mutual understanding of opportunities for achieving SDGs. Yet, we believe that for achieving sustained impact, it is necessary to establish long-term collaborations (**G5**) between application-domain experts and AI researchers and form deep integrated partnerships that allow for enough time to reach good practical solutions^49^.

In interdisciplinary collaborations with a large set of stakeholders, it is important to closely align organisational incentives towards the common goals (**G6**). Taking the involvement of academic researchers as an example, measures of academic success should explicitly take into account the wider societal impact of the work^50^, as citations are known to be a poor proxy for measuring real-world impact^51^.

In some cases, AI4SG collaborations may need to overcome existing organisational barriers to adoption of technology and investment in high-tech solutions (**G7**). Scepticism towards AI is partially rooted in depictions of AI in mainstream media, as well as prior examples of technological solutions that failed to live up to the expectations^52^. Failed attempts to utilise technology for social good come with an associated opportunity cost, given the limited resources available. For effective applications of AI, these barriers will need to be overcome through establishing trust and long-term equal partnerships dedicated to delivering lasting impact.

AI4SG solutions should aim to be cost-effective (**G8**) and this needs to be taken into account early on in the solution design process. There are several ways in which the development cost of AI solutions can potentially be reduced. Skills-based volunteering is a framework through which businesses can enable researchers to offer pro-bono services to NGOs and volunteer for causes that they are passionate about. Hackathons and platforms for crowd-sourcing technical solutions are equally promising, as well as the use of AutoML tools^53^ for automating low-level tasks. Some of AI4SG development costs can be covered by grants, for example those offered by the Google AI Impact Challenge^42^ or the 2030 Vision^54^ aiming to support projects aligned with SDGs.

It is important to be conscious of the different levels of data readiness^55^ across organisations (**G9**) and how they map onto potential ML solutions. Deep learning approaches tend to require large quantities of high-quality data, whereas smaller and noisier datasets may be amenable to exploratory data analysis. Transfer learning and zero-shot learning approaches should be considered in cases where existing trained models can be re-purposed for the relevant use case^56,57^. In the absence of bespoke high-quality data, model development process might benefit from utilising external open datasets like satellite imagery or the existing language corpora and concept ontologies.

Secure data storage, data anonymisation and restricted data access are required to ensure that sensitive data are handled with utmost care (**G10**)^58^. Research data should only include minimal information required to deliver the solution. Responsible information governance should be deeply rooted in respect for human rights, and combined with a high level of physical data security. Data should be encrypted both at rest and in transit. Collaborations should aim to implement existing data governance frameworks for humanitarian action^59^ and consider established standards like the European Union’s General Data Protection Regulation (GDPR) and the United States’ Health Insurance Portability and Accountability Act of 1996 (HIPAA).

## Case studies

Here we highlight three case studies to reflect on how AI4SG collaboration guidelines can be incorporated in mature projects (Troll Patrol), new projects that are just being initiated (Shaqodoon), as well as community-wide initiatives within the AI community aiming to use AI for sustainable development (Deep Learning Indaba).

### Troll Patrol

Having women working in the heart of our democracy is an important step towards achieving gender equality (SDG 5) and strong institutions (SDG 16). This involves creating and protecting inclusive spaces for discussing important political issues. Social media have become an integral part of these conversations and represent an important way of sharing ideas and disseminating information. For women to be equally represented on these digital platforms, they need to be able to share their opinions without fear of abuse.

In Troll Patrol^60,61^, Amnesty International partnered with Element AI’s former AI for Good team to utilise computational statistics and natural language processing methods for quantifying abuse against women on Twitter, based on crowd-sourcing that involved participation of over 6500 volunteers who sorted through 288,000 tweets sent to 778 women politicians and journalists in the UK and USA in 2017. The results of the study have revealed worrying patterns of online abuse, estimating 1.1 million toxic tweets being sent to women in the study across the year, black women being 84% more likely than white women to experience abuse on the platform. The core of the analysis was based on using machine learning approaches to pre-filter the data, followed by applying computational statistics methods. The team has additionally evaluated the feasibility of using a fine-tuned deep learning model for automatic detection of abusive tweets^61^. The evaluation suggests that AI could potentially be used to enrich the work of trained human moderators and make abusive tweet detection easier, despite not being ready to be used without human supervision.

The project involved a deep partnership (**G5**) between an NGO and an AI team, using established methods (**G1, G2**) for a well-defined goal of identifying abusive tweets (**G4**) in order to make digital platforms more inclusive (**G3**). To perform the study, obtaining labelled data was key (**G9**). Given the sensitivity of the data and possibility of increasing exposure to abuse, all study participants were asked if they wanted to remain anonymous in the reports (**G10**). The AI technology developed in the project is not bespoke to tracking abuse against women, making it reusable and of long-lasting value for the team involved in the development (**G6**). Amnesty International having previously engaged with the team on other AI projects helped build trust to make the collaboration possible (**G7**), and brought their deep domain expertise that the quantitative study had complemented. In terms of technical project execution, using a pretrained model from a larger dataset helped reduce the minimum sample size needed for a performant AI abuse detection system, reducing overall costs (**G8**).

### Shaqodoon: AI for improving citizen feedback

Citizens play a pivotal role both in helping deliver on SDGs as well as holding development actors accountable by keeping track of their progress. It is especially important to actively involve communities that may not have the means of making their voice heard. Shaqodoon^62^ is an NGO aiming to improve citizen feedback in Somalia by hosting an interactive voice response platform allowing the citizens to leave feedback on infrastructural projects that affect them. Given that an estimated 65% of the Somali population does not read or write^63^, voice recordings provide an inclusive way of aiming to involve everyone in the conversation (**G3**).

Manually extracting relevant feedback from voice recordings is a laborious process, and Shaqodoon has been looking at ways of using AI for automating the labelling of incoming responses in order to efficiently identify complaints.

Early on in this process, Shaqodoon estimated that there might be up to 80,000 voice recordings available for model development, until a subsequent analysis revealed that only 72 voice recordings had high-quality labels available in an accessible format. This was insufficient for developing an AI solution, making it necessary to reset expectations (**G1**) and improve data readiness (**G9**) through better data collection practices, increasing the number of high-quality labels in a machine-readable format. Shaqodoon worked jointly with ML experts towards identifying the minimum viable AI solution for automated triaging of voice recordings (**G2**) under a formal specification of categories of interest (**G4**), while keeping in mind the privacy of the callers (**G10**). Through this collaboration, Shaqodoon managed to accelerate the project towards the stage where working on AI automation was feasible. The project was selected to be among the finalists of the MIT Solve Challenge^64^, an opportunity for Shaqodoon to obtain resources for model development (**G6, G8**).

### Deep Learning Indaba

Successful implementation of each of our ten guidelines (see Box) relies on bridge-builders who bring disparate AI4SG stakeholders together. These bridge-builders are people who are embedded within local communities, understand development or humanitarian work, and/or have strong technical skills in data science and AI. Our third case study looks at the Deep Learning Indaba^65^, a grassroots organisation that aims to build strong and locally led capacity in artificial intelligence and its applications across Africa. Such organisations can support the realisation of the Sustainable Development Goals by building strong partnerships (SDG 17) and by fostering innovation (SDG 9). Of particular relevance to supporting successful AI4SG outcomes is the ability of such organisations to support greater diversity and inclusion within the field of AI, and in technical communities more generally. Such inclusivity is the best guarantee that AI applications will effectively work towards social good.

The Deep Learning Indaba was established with the mission to strengthen machine learning and AI in Africa, and towards greater self-ownership and self-confidence in AI by pan-African developers and communities. Over the last 3 years, with leadership driven by Africans within their countries and abroad, they contributed to a positive shift in the visibility and ability of Africans in AI. The Indaba, through the critical mass of African AI researchers and engineers it brings together, supports a growing number of AI4SG efforts, including helping to create new datasets like those of African masks^66^, developing new research for language translation^67^, creating new continent-wide distributed research groups to develop on natural language tools for African languages that are often considered ‘low-resourced’^68^, improving outcomes for malaria^69^ and in addressing conservation challenges^70^. The Snapshot Serengeti Challenge^70^ was done in partnership with DeepMind and had involved using thousands of geo-located, time-stamped and labelled images from camera traps in the Serengeti, to develop AI solutions for tracking migration and activity patterns of potentially endangered animal species to help the conservation efforts (SDG 15). The IBM-Zindi Malaria Challenge^69^ was done in partnership with IBM Research Africa and was looking at using reinforcement learning for combining interventions for reducing the likelihood of transmission and reducing prevalence of malaria infections (SDG 3). Groups like the Indaba also work in strong partnership with many other groups, such as Data Science Africa, Black-in-AI and Women in Machine Learning, emphasising the importance of sector-wide collaboration to improve representation. This also makes these organisations better positioned to do justice to the interconnectedness of SDGs. This grassroots approach to building stronger socio-technical communities has also been replicated in other regions, in eastern Europe, south-east Asia and South America, showing the growing ability of global communities in strengthening their own capacities and of their eventual contributions towards AI for Social Good.

Dialogue with technical grassroots organisations like the Deep Learning Indaba informs several of our guidelines: these organisations can ensure inclusive and accessible applications (**G3**); they can create the environment and provide the embedded capacity that supports long-term partnerships (**G5**); they can act as translators between different stakeholders (**G1**); they can help facilitate teams whose work is informed by resource constraints (**G6, G8**) and in need of simple, low-cost solutions (**G2**); they can share the knowledge and experience needed to help establish trust and buy-in necessary for AI4SG collaborations (**G7**). For example, the hackathons that Deep Learning Indaba hosted on AI solutions for conservation and malaria efforts^69,70^ brought together experts from leading centres of research excellence and the local developers to work on finding solutions to important problems of interest to the local community. Industry partners provided high-quality data for these challenges (**G9**) for local developers to find simple prototype solutions (**G2**) in a hackathon format, aimed at reducing development costs (**G8**).

## Call for action

We encourage AI experts to actively seek out opportunities for delivering positive social impact. Ethics and inclusivity should be central to AI systems and application-domain experts should inform their design. Numerous recent advances suggest that there is a huge opportunity for adding value to the non-profit sector and partnerships with NGO experts can help ensure that theoretical advances in AI research translate into good for us all.

We equally encourage all organisations working on sustainable development to consider opportunities for utilising AI solutions as powerful tools that might enable them to deliver greater positive impact, while working around resource constraints by tapping into cost-efficient opportunities, such as skills-based volunteering or crowd-sourcing. To do this with the least amount of friction, we highlighted the need to engage with technical experts early, and to gain insights into prerequisites and feasibility given the level of data readiness. We see the need to create more spaces and opportunities to facilitate partnerships and make it easier to get access to AI expertise.

Given that much of the AI talent is currently involved in industrial or academic research, we would encourage key stakeholders in leading research labs to further empower researchers in donating a percentage of their time to AI4SG initiatives, where appropriate and possible. The complexity of real-world challenges can in fact help to boost the understanding of existing methods and demonstrate impact where it matters the most.

Finally, we invite everyone to join the discussion and help shape the strategy around how to tackle the world’s most pressing challenges with some of the most powerful technological solutions available. Only together can we build a better future.

## Disclaimer

The opinions presented in this paper represent the personal views of the authors and do not necessarily reflect the official policies or positions of their organisations.
